# Thrips Species Composition in Ontario Greenhouse Floriculture: Innovative Identification Tools and Implications for Integrated Pest Management

**DOI:** 10.3390/insects15030211

**Published:** 2024-03-21

**Authors:** Sarah Elizabeth Jandricic, Ashley Summerfield, H. Eric L. Maw, Bryan M. T. Brunet, Rosemarije Buitenhuis

**Affiliations:** 1 Ontario Ministry of Agriculture, Foodand Rural Affairs, Vineland, ON L0R 2E0, Canada; 2 Vineland Research and Innovation Centre, Vineland, ON L0R 2E0, Canada; ashley.summerfield@vinelandresearch.com (A.S.); rose.buitenhuis@vinelandresearch.com (R.B.); 3 Ottawa Research and Development Centre, Agriculture and Agri-Food Canada, Ottawa, ON K1A 0C6, Canada; eric.maw@agr.gc.ca (H.E.L.M.); bryan.brunet@agr.gc.ca (B.M.T.B.)

**Keywords:** thrips, *Thrips tabaci*, pest management, species identification, floriculture, ornamentals, greenhouse, illustrated key

## Abstract

**Simple Summary:**

Thrips are minute insects that, despite their small size, are one of the most damaging and challenging pests affecting ornamental crops. Ontario growers have observed a recent increase in thrips damage that was not adequately controlled by standard biological control programs developed for the primary pest species, Western flower thrips (*Frankliniella occidentalis* (Pergande)). This led to an assumption that biological control programs were no longer effective or suffered from quality control issues. Proper species identification is critical for successful pest management; however, efforts to identify thrips species in Canadian greenhouses have not been formally made since the 1980s. We sampled thrips communities from eight commercial floriculture greenhouses in the Niagara region (Ontario, Canada) from 2016 to 2019. A secondary species, onion thrips (*Thrips tabaci* Lindeman), constituted a large percentage of the species found in ornamental crops and appears to be largely responsible for increased crop damage. The recent interception of other exotic thrips species found in ornamental crops further heightens the need for prompt identification when unusual damage or outbreaks occur. As traditional taxonomic keys are inaccessible to non-specialists due to their technical difficulty, we developed a simplified identification key for use by growers and IPM consultants.

**Abstract:**

Proper species identification is the keystone of successful integrated pest management (IPM). However, efforts to identify thrips species in Canadian greenhouses have not been formally made since the 1980s. In response to recent increases in crop damage, we sampled thrips communities from eight commercial floriculture greenhouses in the Niagara region (Ontario, Canada) from May until August 2016. Selected sites were revisited in 2017, 2018, and 2019 to determine changes in species composition over time. Western flower thrips (*Frankliniella occidentalis* (Pergande)), along with onion thrips (*Thrips tabaci* Lindeman), constituted the majority of species found. Other pest species (less than 8% of specimens across all sampling years) included poinsettia thrips (*Echinothrips americanus* Morgan), chrysanthemum thrips (*Thrips nigropilosus* Uzel), and *Frankliniella fusca* (Hinds). Further investigations of thrips outbreaks in Ontario from 2016 to 2023 revealed other important species, including *Thrips parvispinus* (Karny), *Hercinothrips femoralis* (Reuter), and *Scirtothrips dorsalis* Hood. The current biocontrol strategies used in Ontario floriculture crops for western flower thrips do not adequately control onion thrips or other thrips pests in ornamental crops, making identification a fundamental step in determining whether biocontrol or chemical control strategies should be implemented. However, traditional taxonomic keys are inaccessible to non-specialists due to their technical difficulty. Using the data gathered in these surveys, we developed a simplified, illustrated identification key for use by growers and IPM consultants.

## 1. Introduction

Thrips (Thysanoptera) are one of the most destructive crop pests worldwide [1]. They can reduce yield via direct feeding damage to foliage [2,3] and the transmission of tospoviruses, such as Impatiens necrotic spot virus (INSV) [4,5] and Tomato spotted wilt virus (TSWV). Ornamental crops especially have a low threshold for thrips and their damage for aesthetic reasons; thrips feeding generally causes unsightly “silver” patches on foliage and streaking of petal tissues that are unacceptable to consumers [6] and can cause the deformation of leaf tissue. Thus, thrips management is currently a leading priority for floriculture growers in Canada [7].

Since its global range expansion in the 1980s, western flower thrips (WFT), *Frankliniella occidentalis* (Pergande), has been the primary thrips pest in greenhouse floriculture crops around the world [8], including Canada [9]. Although WFT populations tend to be resistant to all chemical control options currently registered by Health Canada, this thrips has been generally well managed in greenhouse floriculture crops in Canada since the early 2000s using comprehensive biological control programs [9]. However, a growing number of unmanaged thrips outbreaks and unusual damage patterns have been seen in greenhouse ornamentals in the large production region of Southern Ontario starting in 2012 [10]. 

Other greenhouse floriculture pests that are larger and have limited species compositions in greenhouses in northern latitudes, such as aphids (four major species) and whiteflies (two major species), can be distinguished by growers and consultants using hand lenses (15–20× magnification). Many extension factsheets have been created over the years for these purposes [11,12]. However, partially due to their small size, thrips species identification cannot be conducted on-site by growers or consultants with such simple methods. Because of this and the ubiquitous prevalence of WFT, little effort has been made to confirm the species responsible for these new outbreaks, and WFT was assumed to be the pest species responsible when light-coloured thrips were present. Subsequently, biocontrol failure was suspected to be the cause of the outbreaks, even by growers that had very robust WFT biocontrol programs that had been working consistently for years. Measures were taken to confirm the quality of natural enemies [13], and this was proven to be sufficient. It was then suspected that novel thrips species may be present in ornamental crops that are less controlled by current management programs. 

Onion thrips (OT), *Thrips tabaci* Lindeman, has been suspected as a possible emerging species in floriculture crops [10] due to its prevalence as an outdoor pest [14,15] and similar appearance to WFT. This species was formerly the main thrips pest in both vegetable and floriculture greenhouses in Canada before being supplanted by WFT in the 1980s [16,17]. Onion thrips has persisted in Canada as an important pest of outdoor vegetable crops such as onions, leeks, and cabbage [18]. Onion thrips has been established throughout North America for at least the last century [19], and WFT, which is originally native to the Southwestern USA, has been established as a greenhouse pest in the northeast of North America for nearly 40 years [8]. However, there are few published data that describe the prevalence of these species within Canadian greenhouses. After WFT were first detected in Ontario, the largest floriculture production area in Canada, Broadbent et al. [17] conducted surveys in and around greenhouse floriculture crops from 1983 to 1986. At that time, three sites had exclusively OT, and WFT were found in 20 of the 23 Ontario greenhouses surveyed. However, the relative species proportions at these sites were not reported, and no further greenhouse surveys have been published. 

Given that proper pest identification is a keystone of IPM theory [20] and essential to the success of IPM programs, the need for more current and detailed thrips survey data in Ontario is long overdue. Ontario has remained among the top four states/provinces producing the highest farm-gate sales of greenhouse floriculture crops in North America for the past 20 years [21]. As such, it plays a pivotal role in providing accurate IPM information to the greater North American ornamental industry. Given that current biocontrol strategies may not adequately control thrips pests other than WFT in ornamental crops, proper identification is a fundamental step in determining whether biocontrol or chemical controls should be implemented by growers in the short term. In the long term, these data can serve to guide research in Ontario toward effective non-chemical programs for other prevalent thrips species besides WFT. Thus, the primary objectives of this research were to (1) characterize the species composition of thrips in Ontario floriculture greenhouses, (2) to develop a simplified key to thrips species that could be used in the northeast of North America by growers and IPM professionals based on the species found, and (3) to highlight the greater implications of emerging thrips species on IPM programs for greenhouse ornamentals.

## 2. Materials and Methods

### 2.1. Thrips Collection

In 2016, thrips species were sampled every 2–4 weeks at 8 commercial floriculture greenhouses in Southern Ontario starting in May or June (depending on when access was granted and crop availability) for a total of 4–5 sampling dates per site. Thrips sampling began in May when temperatures were consistently warm enough outside (>15 °C) to ensure that we were capturing the presence of any flying thrips populations [22], which could enter the greenhouse through vents, and continued through the season in which growers most struggle with thrips—generally until late August [23]. 

All sites sampled were located in the growing region of Niagara, which holds the highest density of floriculture growers in Canada, with the exception of one site located in the neighbouring municipality of Hamilton. At some sites, multiple compartments within the same greenhouse were sampled if they held a different crop. The sites, crops, and years sampled are detailed in Table 1. All facilities managed their pests predominantly through biological control, with the limited use of pesticides for non-thrips pests as needed (e.g., aphids, spider mites).

We sampled a mix of potted and cut-flower crops of economic importance in the region. Potted crops generally have 8–12-week crop cycles, and all plant stages are present in the greenhouse at once to provide a non-stop supply of flowering plants to customers. Plants producing cut flowers can remain in the greenhouse for much longer (up to 3 years for cut gerbera) but continuously produce flowers that are harvested weekly. Because large greenhouse floriculture operations never have periods of dormancy, and thrips have continuous, overlapping generations within the protected environment of the greenhouse, all life stages of any thrips species could be expected on any sampling date. This is especially true as populations could be a result of being brought in on cutting material early in propagation [9], fly-ins from outside, or carry-overs from either of these avenues. However, we only collected adult thrips for ease of identification. 

In 2016, each crop was sampled evenly across all benches/rows to ensure a representative sample of all plant varieties present had thrips collected from them. Crops were sampled using a “plant tapping” method, in which plant foliage and flowers are briskly tapped over a white tray to dislodge insects. Adult thrips were collected from trays with an aspirator and transferred to vials filled with 90% ethanol for later identification. Both vegetative and flowering plants were captured on all of our sampling dates (with the exception of very small plants that were difficult to tap). Those performing the sampling spent roughly an hour collecting in an attempt to obtain a minimum of 50 specimens per crop and thereby ensure that species proportions could be assessed. As thrips pressure is relatively low in ornamental crops compared to other cropping systems, this meant that at least 5% of the crop on each bench in a compartment had to be tapped in an attempt to reach this threshold. Due to resource limitations, we discontinued sampling if two people had been sampling the same crop for 1 h and had not reached 50 thrips (which was often the case). Where higher thrips pressure was present (e.g., cut-flower crops), we increased the minimum threshold to 200 adult thrips in an hour to ensure sampling across the entire crop was consistent. This sampling method led to 3–200 adult thrips being collected per crop, per date. Crops included potted and cut chrysanthemums (*Chrysanthemum indicum* L.), gerbera *(Gerbera jamesonii* Bolus ex Hooker f.), ivy geraniums (*Pelargonium peltatum*. L’Hér.), New Guinea impatiens (*Impatiens hawkeri* W.Bull), and tropical plants, including hibiscus *(Hibiscus rosa-sinensis* L.) and mandevilla *(Mandevilla splendens* (Hook.f.) Woodson).

To help characterize thrips species composition in economically important crops over time, a subset of commercial greenhouses growing gerbera and chrysanthemum were revisited in subsequent years (Table 1). Two potted chrysanthemum operations were sampled again in July and August of 2017 to assess summer thrips communities (i.e., those potentially resulting from invading thrips species from outside), and one potted gerbera site was sampled once in 2017. Three sites (one potted chrysanthemum, one cut chrysanthemum, one cut gerbera) were also revisited 3 times in the fall (i.e., between 21 September and 21 December), once in October, November, and December of 2018. This was to determine whether non-WFT thrips species were persisting in greenhouses where OT populations had occurred over several summers, but after invasion from outside was no longer possible. In October in Ontario, temperatures are generally below the thermal threshold for thrips flying [22] (see Results Section for specific recorded temperatures demonstrating this). One potted chrysanthemum site was also revisited in 2019 as part of a separate year-long study [24]. Only the species composition data from May to August have been included here, as they correspond to both the original 2016 survey period and the 2019 period.

In addition to formal thrips surveys, local growers and IPM consultants often submit thrips specimens for identification to the Ontario Ministry of Agriculture, Food and Rural Affairs (OMAFRA) when they are experiencing an outbreak or seeing unusual damage symptoms. The instances in which thrips were identified as species other than WFT were recorded and tracked to help further characterize these more unusual thrips outbreaks. 

### 2.2. Thrips Identification and Development of a Simplified Key

The initial thrips species determinations in 2016 were made using multiple morphological keys [25,26,27,28,29]. Subsequently, these resources were used to create a simplified morphological key (see Section 3.5) where commonly occurring species could be more easily identified using a stereomicroscope at 40× magnification. Uncommon species (e.g., those intercepted in Ontario on imported plant material), and a subset of OT and WFT samples, were further examined under a compound microscope to confirm species identifications using structures visible at 100× magnification. The simplified key and a subset of all species identifications were verified by EM and BB at the Canadian National Collection of Insects Arachnids and Nematodes (CNC; Ottawa, ON, USA). Voucher specimens are retained here and at the Vineland Research and Innovation Centre in Vineland, ON. The development of a key using more prominent features meant that the slide mounting of thrips was generally not necessary, and a dissecting microscope could be used in most cases, which many greenhouse staff, consultants, and IPM professionals own or have access to in Ontario. Images used in the illustrated key were created using a combination of Affinity Designer (Serif (Europe) Ltd., Nottingham, UK) and Inkscape (Inkscape Project, 2020. Inkscape, available at https://inkscape.org, (last accessed on 1 February 2024)). The ease of use of the simplified key was validated through grower testing and workshops in Ontario. An early version of this key was made available to growers in October 2018 via the ONfloriculture blog [30] and has since been revised based on user feedback and new species identified during the course of this research. For further reference, colour micrographs of all species included in the key are included in our “Thrips Identification Workshop Guidebook” under the “Thrips Identification” tab on the ONFloriculture blog.

### 2.3. Data Analyses

As the goal was to collect as many thrips species as possible on each sampling date in order to take a broad look at thrips issues in the industry, sampling was not standardized (e.g., by plant number). Thus, proportions, rather than numbers, are expressed. To determine how thrips species composition differed between crop types over the summer in Ontario, data were pooled across sampling dates. In cases where the same crop was sampled across multiple greenhouse sites, the average proportion (±SE) across locations is presented. As this is a descriptive study, no statistical analyses have been conducted on these data. 

## 3. Results

### 3.1. Thrips Species Found in Floriculture Greenhouses in Ontario in Summer 2016 and 2017

In 2016, a total of 2663 thrips were collected and identified. Only five species of thrips were present: *F. occidentalis* (Pergande) (western flower thrips, WFT), *T. tabaci* Lindeman (onion thrips, OT), *Echinothrips americanus* Morgan, and a low percentage of *Frankliniella fusca* (Hinds) and *Anaphothrips obscurus* (Müller). *Anaphothrips obscurus*, unlike the other species, is not considered a pest of ornamental crops (but rather, of cereal crops) and may have blown in from the surrounding fields. 

In most crops, WFT made up the majority of the thrips population (Table 2). However, OT accounted for more than 25% of the thrips collected from gerbera crops (both potted and cut) and two of the three potted chrysanthemum sites. Onion thrips were also present in ivy geranium, hibiscus, mandevilla, and impatiens, though at generally low levels (<20%). 

The prevalence of OT varied considerably between crops within the same site. For example, Site 2 had a very high proportion of OT in potted gerbera (93%), but an extremely low incidence of OT in mandevilla (<1%) in an adjacent compartment (Table 2). A similar phenomenon was observed at Site 6, where cut gerbera had an average of 35% OT and a maximum proportion of 76% over all sampling dates, while cut chrysanthemums in an adjacent compartment at this site had less than 14% OT on all sampling dates.

### 3.2. Thrips Species Composition in Greenhouses Revisited in Summer 2017 and 2019

**Figure 1:**
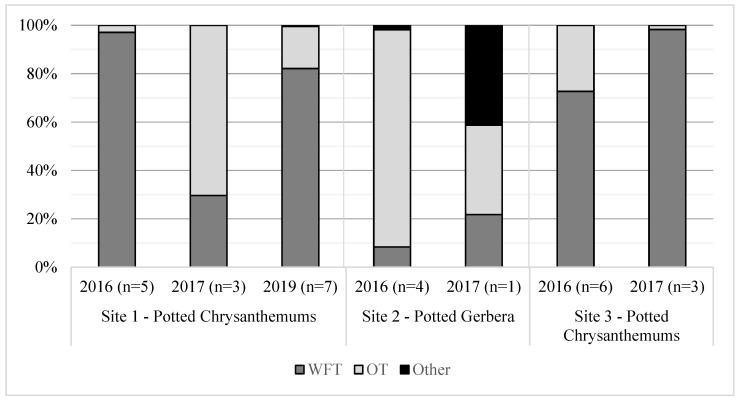
Relative proportions of *Frankliniella occidentalis* (WFT), *Thrips tabaci* (OT), and other thrips species at 3 commercial greenhouses over three summers. Number of sampling events per site are listed in brackets for each year. Sites were sampled from May to August in 2016 and 2019 and from July to August in 2017. *Echinothrips americanus* accounted for the majority of “Other” thrips at Site 2 in 2017.

Sites sampled in subsequent years revealed that OT were continuously present, but with large variations in relative species proportions between sampling years (Figure 1). Variations were not consistent between sites. Specifically, at Site 1, OT made up a small proportion of thrips in 2016 but the majority of thrips in 2017, while at Sites 2 and 3, there was a greater proportion of OT in 2016 compared to 2017.

### 3.3. Thrips Species Composition in Greenhouses Revisited in Fall 2018

In fall 2018, revisits to sites known to have OT populations in previous summers revealed that OT can persist in floriculture operations after fly-ins from outside are no longer possible. WFT and OT are not able to fly at temperatures below 15–17 °C [22,31]. The outside recorded max. daily temperatures did not exceed 17 °C from 12 October 2018 (2 weeks before sampling date), to 14 December 2018 (final sampling date), and only exceeded 15 °C on three occasions (15.8, 15.6, and 16.6 °C on 14 October, 19 October, and 6 November). the mean temperature for the study period was 3.8 °C [32]. At all sites, OT were present on all three sampling dates, demonstrating that these thrips had become established in these crops. Species ratios and the occurrence of other thrips species varied by location. Site 1 (potted chrysanthemums) had an average of 58% (±5) OT over the three sampling periods, with 29% (±14) WFT, 11% (±10) *Thrips nigropilosus* Uzel, and 2% *Frankliniella fusca* (*N* = 147 thrips total). Site 6 (cut gerbera) had 55% (±15) WFT, 41% (±14) *E. americanus*, and 4% (±1) OT (*N* = 370 thrips). Site 5 (cut chrysanthemums) had the lowest number of detectable OT in fall 2018, with 91% (±5) WFT, 7% (±4) *T. nigropilosus*, and 2% OT (±1) (*N* = 150 thrips total).

### 3.4. Thrips Populations or Outbreaks Identified after 2016 in Ontario 

Since the start of this survey, a number of other species have caused outbreaks or unusual damage in greenhouse ornamentals in Ontario (Table 3). Six outbreaks of OT occurred, including two outbreaks in cyclamen at separate facilities, both being grown at relatively cool temperatures (12.5–15 °C). Tropical foliage was frequently a source of unusual thrips activity. *Bagnalliella yuccae* (Hinds), *E. americanus, Frankliniella schultzei* (Trybrom)*, Hercinothrips femoralis* Reuter, OT, and *Thrips parvispinus* (Karny) have been identified on popular tropical plants. *Thrips setosus* Moulton was intercepted on Hydrangea. Further details on the interceptions of *T. parvispinus* and *T. setosus*, two species new to Ontario, can be found in Gleason et al., 2023 [33].

### 3.5. Simple Key to Thrips Species Found in Ontario Greenhouses for Growers 

This simplified key was designed for use by growers and IPM practitioners in greenhouse floriculture and vegetable crops in Canada. The key presented here has been shortened to include only the necessary illustrations. The full version includes illustrations depicting identification features for each step and can be found at https://onfloriculture.com/simple-thrips-id-key/ (last accessed on 1 March 2024). 

This key is not comprehensive for all thrips species that may occur in North America but includes those most likely to be encountered in floriculture and/or vegetable greenhouses in Canada and/or the Great Lakes region of North America. The key also includes species that may be intercepted on imported plant materials, based on our thrips identifications on plant material in Ontario over the 7 years covered in this paper (2016–2023).

**Figure 2:**
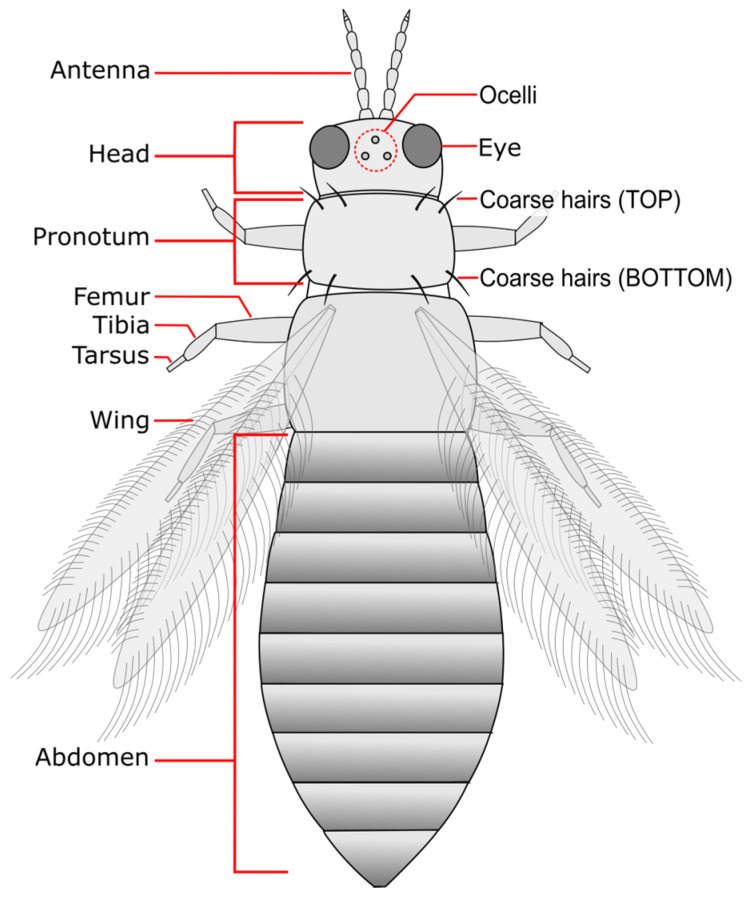
General anatomy of an adult thrips and features used for identification in this key. Specimens should be positioned with their back and wings facing toward the viewer.

All features used in this key (Figure 2) are found on the dorsal side (with wings facing toward the viewer) and can typically be seen using a mid-quality dissection microscope that has a maximum magnification level of at least 40×. In formal taxonomic keys, the terms anterior/posterior and marginal/angular would be used to indicate the position of hairs on the pronotum. However, for ease of understanding by those not formally trained in insect identification, we have replaced these terms with “top/bottom” and “inner/outer”, respectively.

Some advanced identification features requiring a compound microscope, which may be useful in identifying potential exotic species, can be found in the complete version of the key.

**Figure 3:**
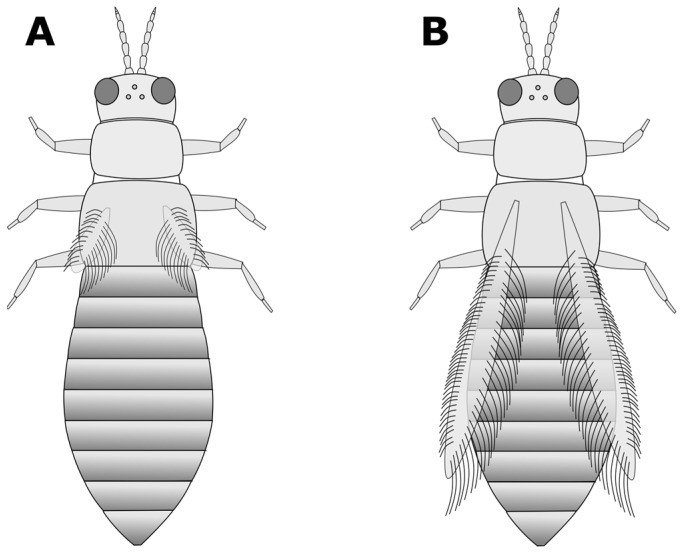
Step 2—adult thrips with (**A**) short wings and (**B**) long wings.

**Figure 4:**
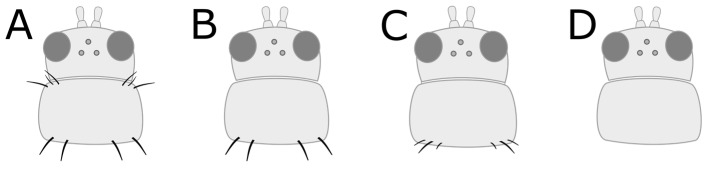
Coarse hairs on the pronotum. (**A**) Long hairs on top and bottom, (**B**) long hairs on bottom only, (**C**) short hairs on bottom, (**D**) no coarse hairs anywhere on pronotum.

**Figure 5:**
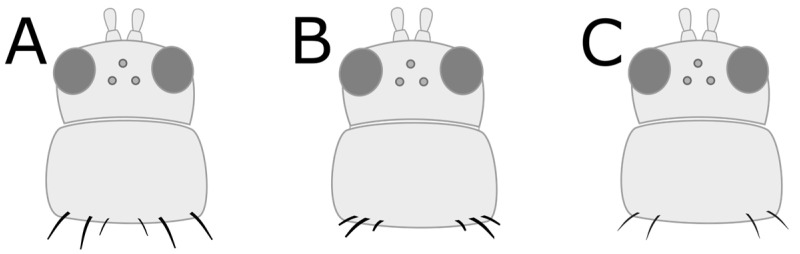
Coarse hairs on bottom of pronotum: (**A**) three pairs, outer two hairs are long and of equal length, innermost hairs are shorter; (**B**) three short pairs, second pair distinctly longer than the other two, and (**C**) two short pairs that are relatively fine and therefore often difficult to see under a dissection microscope.

**Figure 6:**
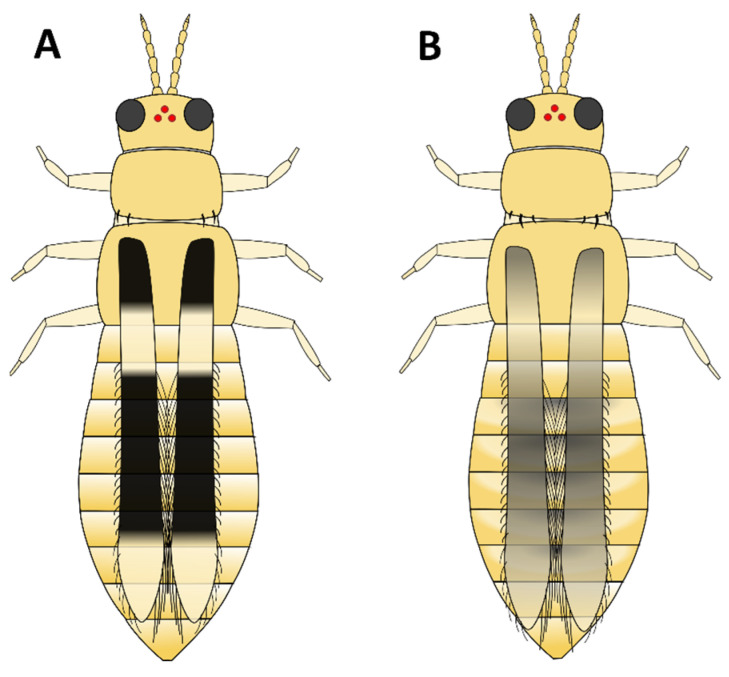
Wing patterns of (**A**) *Chaetanaphothrips orchidi* and (**B**) *Scirtothrips dorsalis*.

**Figure 7:**
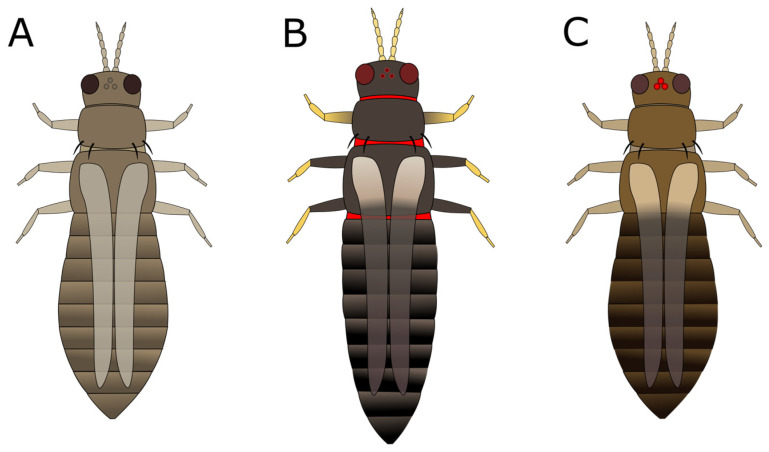
Body and wing patterns of (**A**) *Thrips tabaci*, (**B**) *Echinothrips americanus*, and (**C**) *T. parvispinus* or *T. setosus*.

**Figure 8:**
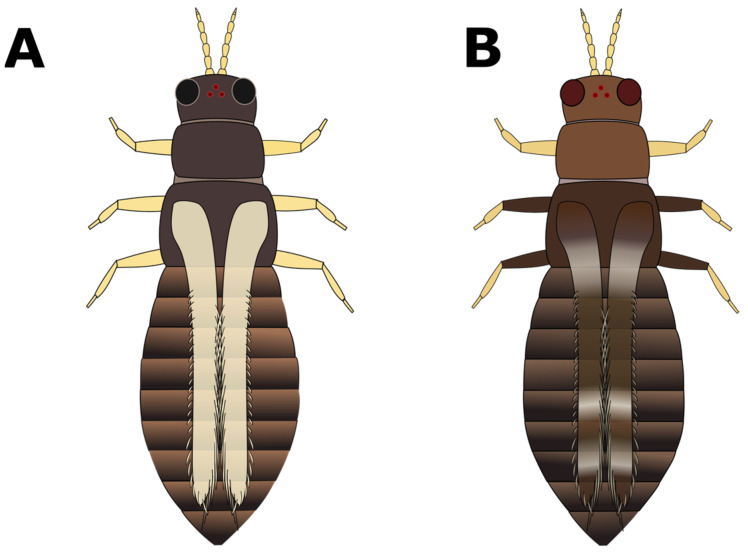
Leg, wing, and body colour patterns of (**A**) *Heliothrips haemorrhoidalis* and (**B**) *Hercinothrips femoralis*.

1. a. Head and pronotum tan or yellow; abdomen tan, yellow, or light to medium brown (GO TO STEP 2).
  b. Head, pronotum, and abdomen brown to black in colour; head and pronotum may be lighter brown than abdomen, but not yellow (GO TO STEP 7).
2. a. Very short wings, shorter than the width of the body (Figure 3A); foliar feeding damage typically on lower leaves, usually only found on chrysanthemums and gloxinias:
  ***Thrips nigropilosus***, “chrysanthemum thrips”, wingless form.
  (Note: both winged and wingless forms may be present in the same population).
  b. Long fringed wings extending nearly the full length of the body (Figure 3B) (GO TO STEP 3).
3. a. Pronotum has two pairs of long coarse hairs on both the top and bottom of the pronotum (Figure 4A); ocelli are red:
  ***Frankliniella occidentalis*** “western flower thrips” (most common).
  (May also be other *Frankliniella* species, such as *F. tritici, F. bispinosa*, or *F. schultzei* (pale forms). Differentiating between *Frankliniella* species requires a compound microscope and advanced identification skills.)
  b. No long coarse hairs on the top of the pronotum. There may be hairs on the bottom edge of the pronotum; ocelli may or may not be red (Figure 4B–D) (GO TO STEP 4).
4. a. Ocelli are grey; two pairs of long coarse hairs on the bottom edge of the pronotum (Figure 4B):
  ***Thrips tabaci***, “onion thrips”.
  b. Ocelli are red; coarse hairs on the bottom of the pronotum may be long or short (Figure 4B–D) (GO TO STEP 5).
5. a. Three pairs of coarse hairs on the bottom of the pronotum, outer two pairs of hairs of equal length, which are distinctly longer than the inner pair (Figure 5A); wings are pale or absent; foliar feeding damage, typically on lower leaves; usually only found on chrysanthemums and gloxinias:
  ***Thrips nigropilosus***, “chrysanthemum thrips”, winged form.
  (If found on crops other than chrysanthemum and gloxinia, it may be *T. palmi,* which is not present in Canada but may be intercepted on plant material imported from tropical regions, including Florida, Mexico, and Central America. Differentiating between *T. nigropilosus* and *T. palmi* requires a compound microscope and advanced identification skills. It could also be male *T. parvispinus* or *T. setosus*: see Step 10 for identification of females if dark-coloured thrips are also present.)
  b. Coarse dark hairs on the bottom of the pronotum are short and may be difficult to see (Figure 5B,C); small body size compared to other common thrips species; wings are grey or have black markings; usually found on tropicals (GO TO STEP 6).
6. a. Head and body yellow; wings black with distinct pale band in the middle (Figure 6A); hairs on the bottom of the pronotum are short and fine and often difficult to see with a dissection microscope (Figure 5C); usually found on tropicals:
  ***Chaetanaphothrips orchidii***, “orchid thrips”.
  (This species is not present in Canada but widespread in tropical and sub-tropical regions, including California and Florida; it may be intercepted on imported plant material.)
  b. Head and body yellow; wings are grey with some paler sections but not distinctly banded (Figure 6B); coarse dark hairs on the bottom of the pronotum are short, middle pair distinctly longer than the others (Figure 5B) (may be difficult to see depending on the quality of your microscope); usually found on tropicals; distinctive feeding damage causes distortions at growing points, resembling broad mite damage:
  ***Scirtothrips dorsalis***, “chilli thrips”.
  (This species is not present in Canada, but present in Florida, Texas, Mexico, and the Caribbean; it may be intercepted on imported plant material.)
7. a. Pronotum has long coarse hairs (Figure 4A–C). Note: hairs may be difficult to see on black thrips—may be easier to see in side-view (GO TO STEP 8).
  b. No long coarse hairs on the pronotum (Figure 4D); front legs entirely yellow (GO TO STEP 11).
8. a. Long coarse hairs on both top and bottom of pronotum (Figure 4A); no red pigment visible between the segments; wings uniformly pale brown:
  ***Frankilinella fusca***, “tobacco thrips”, winged form (most common).
  (May also be *F. occidentalis*, dark morph (usually seen in fall and winter), *F. intonsa* (not known from Ontario, but present in British Columbia), or *F. schultzei* (not present in Canada but present in Florida, Central America, and the Caribbean, may be intercepted on imported plant materials)).
  b. No long coarse hairs on top of pronotum, but bottom has two pairs of long coarse hairs (Figure 4B); red pigment may or may not be visible between segments; wings may be uniformly pale or dark with pale bands (GO TO STEP 9).
9. a. Head and body light to medium brown, never black, uniform in colour; grey ocelli; wings uniformly pale in colour (Figure 7A):
  ***Thrips tabaci***, “onion thrips”, dark morph.
  b. Abdomen dark brown to black; head and pronotum either medium brown or black; red ocelli; light patches at the top of the wings (visible on dry specimens) (Figure 7B,C) (GO TO STEP 10).
10. a. Head and body black; red ocelli (may be difficult to see); red pigmentation often visible between body segments; back legs yellow with black femurs, femurs on front legs dusky at base but not distinctly black; abdomen widest at the top and narrowing toward tip (Figure 7B):
  ***Echinothrips americanus***, “poinsettia thrips” (most common).
  (*Dichromothrips corbetti* is similar in appearance to *Echinothrips*, but is only present on orchid crops, and does not have hairs on the top or bottom of the pronotum.)
  b. Head and pronotum medium to dark brown, abdomen darker than head; ocelli bright red and easily visible; no red pigmentation between segments; abdomen widest in the middle (Figure 7C):
  ***Thrips parvispinus***, “pepper thrips”, females, or ***Thrips setosus***, “Japanese flower thrips”, **females**.
  (The males of both species are pale yellow. Neither species is established in Canada but present in some US states; they may be intercepted on imported plant material. In northern latitudes, *T. parvispinus* is typically intercepted on tropicals, and *T. setosus* has been found on Hydrangea. Differentiating between these two species requires a compound microscope and advanced identification skills.)
11. a. All legs entirely yellow; head and pronotum as dark as or darker than the abdomen; wings uniform in colour and paler than the body (visible on dry specimens) (Figure 8A):
  ***Heliothrips haemorrhoidalis***, “greenhouse thrips”
  b. Front legs yellow, brown femurs on back legs; head and pronotum often paler than abdomen; light bands at the top and tips of the wings (visible on dry specimens) (Figure 8B):
  ***Hercinothrips femoralis***, “banded greenhouse thrips”.

## 4. Discussion

Although our survey represents a small portion of Ontario production and a limited number of crops compared to the wide variety represented in this sector, it provides a useful snapshot of the current state of thrips communities in greenhouse floriculture crops. More surveys and increased monitoring of thrips communities are needed in a variety of greenhouse crops and growing regions to form a truly comprehensive understanding of how the species composition is changing. Future studies should take care to standardize the sampling methods, locations, and timing at all sites to better compare thrips diversity and species. 

What is clear, however, is that the thrips species composition in greenhouse floriculture crops has changed in the decades since the last survey was conducted. These results confirmed the suspicion that western flower thrips (*Frankliniella occidentalis* (Pergande); WFT), while still the most prevalent, is no longer the only thrips species affecting greenhouse ornamentals. Onion thrips (*Thrips tabaci* Lindeman; OT) was the second most prevalent species in our detailed surveys from 2016 to 2019, making up approximately one-third of all specimens collected across all crops sampled. Further, investigations of unusual thrips or thrips damage from 2016 to 2024 revealed a total of 11 other pest thrips species—far more than we realized were present in Ontario floriculture crops. 

Our results support the few recently published thrips surveys in horticultural crops. A French study reported WFT as the dominant pest, but with OT comprising 42% and 18% of thrips collected in rose greenhouses in 2007 and 2008, respectively [34]. Another French study using molecular methods confirmed that OT was the primary pest thrips species in ornamental greenhouses, accounting for 47% of the population [35]. Mixed thrips communities of WFT, OT, and other thrips species have also been recorded in high-tunnel strawberries in the UK [36] and Denmark [37]. Similar to our results, Japanese researchers were surprised to discover that *Thrips palmi* Karny, the species previously assumed to be dominant in field-grown chrysanthemum in Okinawa, were far outnumbered by chrysanthemum thrips (*T. nigropilosus* Uzel) [38]. These findings suggest that mixed thrips species populations could be a common occurrence in horticultural crops worldwide and not an isolated phenomenon affecting Ontario.

The status of OT as a common outdoor pest in Ontario [18] points to outdoor populations (“fly-ins”) as the most likely source of infestation in greenhouse crops. Although this cannot be confirmed by the results of this study, their presence in multiple crops at all study sites and in all years suggests that this pest is ubiquitous in the landscape in the Niagara region of Ontario. If fly-ins are the primary infestation source, this may explain some of the variation observed between years and locations. Weather patterns such as precipitation, temperature, and wind speed can all impact outdoor thrips population size and/or behaviour [31,39,40]. Land use patterns also contribute to variability between sites. A study of Hungarian pepper greenhouses found that OT was dominant in rural greenhouses, whereas WFT was dominant in urban greenhouses [41]. Future surveys should incorporate outdoor sampling and standardized collection practices designed to quantify thrips at each sampling event, which may provide further insight into the sources of and seasonal variation in thrips invasion.

Onion thrips were also found in our study sites in the fall, after temperatures were below the thermal flight threshold [31,42] and therefore too cold for outdoor populations to be an infestation source. This strongly indicates that OT are able to establish long-term populations within greenhouse ornamental crops. Alternatively, imported plant material could be acting as a secondary source of infestation in some crops. Research by Buitenhuis et al. [43] found that imported chrysanthemum cuttings frequently contained thrips. Although the thrips collected from the cuttings were presumed to be WFT, species identifications were not verified using morphological keys, and it is therefore possible that chrysanthemum cuttings could also be a source of OT through the fall and winter months. Further research is needed to confirm the relative impact of different infestation sources so that growers can take steps to reduce pest pressure. 

Although fly-ins affect the number of thrips entering the greenhouse, we also hypothesize that IPM decisions likely influence thrips species proportions that become established in the crop. Insecticides are the primary method of thrips control in most agricultural cropping systems [44]. But, since, historically, intensive pesticide use led to extensive insecticide resistance in *F. occidenatlis* in greenhouse crops [45,46,47], biological control is now the primary thrips management strategy in many floriculture greenhouses worldwide. This most often includes regular applications of predatory mites (Acari: Phytoseiidae and Hypoaspidae) and nematodes (Rhabditida: Steinernematidae), repeated applications of microbial pesticides (Ascomycota: Hypocreales), and the occasional use of true bugs like *Orius* (Hemiptera: Anthocoridae) [9,16,48].

The use of strictly biocontrol tactics may promote the establishment and proliferation of OT, as well as other thrips species, in some greenhouses. Conversely, greenhouse IPM programs incorporating more broad-spectrum chemical pesticides for other pests may accidentally be controlling OT in some facilities, shifting population proportions toward WFT, which are more insecticide-resistant. Fitness trade-offs of insecticide resistance have been well documented in many pest species, including thrips [49,50,51,52,53,54]. Under experimental conditions, Zhao et al. [55] found that a resistant population of WFT outcompeted OT in the presence of pesticides, but OT became dominant when no pesticides were applied. Insecticide resistance is a key trait that contributed to WFT’s status as the foremost pest thrips in greenhouses [8,56,57]. It is therefore reasonable to suspect that the shift away from pesticides toward biocontrol-based IPM is contributing to the increased prevalence of non-WFT species. Regions with a high rate of biocontrol adoption, such as Ontario, may therefore be more likely to experience greater thrips species diversity.

Of greatest concern to growers is the fact that higher proportions of OT seem to be associated with increased plant damage, and that these thrips outbreaks are unmanaged by typical biocontrol tactics (personal observations, S. Jandricic). While most biocontrol products have been tested for OT individually [58,59,60], the comparative efficacy of many greenhouse biocontrol tactics on OT vs. WFT is currently unknown (efficiency and preferences of various predatory mites, efficacy of microbial-based products, etc.). Research into the relative efficacy of commonly used biocontrol agents may elucidate the reasons why current IPM strategies designed for WFT fail to adequately control OT in floriculture operations. Until more effective biocontrol-based management strategies can be developed, growers with a high proportion of OT will continue to rely on pesticides to manage outbreaks. Similar outcomes are currently being seen with other thrips species found in this survey. Pests like *Thrips Parvispinus* Karny, *Echinothrips Americanus* Morgan, and *Hercinothrips femoralis* (Reuter) are proving difficult or expensive to control with biocontrol tactics, making chemical applications necessary when outbreaks occur (personal observation, S. Jandricic).

The development of the simplified key to identify thrips found in greenhouses allows growers to make judicious use of such pesticide applications. Since WFT are generally resistant to all available products in Canada, it is critical that growers properly identify which thrips species are in their crops. Applications of chemical pesticides to a primarily WFT population are unlikely to result in meaningful pest reductions and could interfere with existing beneficial insects established in the crop [61,62,63,64]. In such cases, the best course of action is to introduce additional biocontrol products, increase the release rates of existing ones, or add physical controls like mass trapping [23]. While negative impacts on beneficials are equally possible when treating for OT or other pest thrips, the higher likelihood of successful pest reduction may be sufficient to offset those risks. 

Overall, the results of our survey demonstrate that the thrips species composition is more variable in ornamental greenhouses than initially assumed. Additionally, this seems to be increasing since this survey was first started in 2016, with more reports of invasive or emerging thrips species occurring in Ontario. This may be due in part to the increased trade of plant material, a shift in crops grown (e.g., toward more tropical ornamental plant species), and/or the effects of global warming, allowing pests to expand their ranges [65]. Increased grower awareness due to these survey results being presented via various forums in Canada and the development and use of the thrips key for growers may also be contributing to the increase in uncommon thrips species being found. For growers to make accurate and timely pest management decisions, surveys and tools such as those presented in this paper are encouraged for other floriculture regions.

## Figures and Tables

**Table 1:** Number of sampling dates per crop at commercial greenhouses (site) in Ontario from 2016 to 2019. Crops were potted unless otherwise specified.

Site	Crops Sampled	2016	2017	2018	2019
1	Chrysanthemums	5	3	3	6
2	Gerbera	4	1		
	Mandevilla	3			
3	Chrysanthemum	6	4		
	Hibiscus	7			
4	Gerbera	3			
5	Cut Chrysanthemums	6		3	
6	Cut Chrysanthemums	5			
	Cut Gerbera	4		3	
7	Ivy Geranium	2			
	New Guinea impatiens	4			
8	Chrysanthemums	2			

**Table 2:** The relative proportions of *Frankliniella occidentalis* (WFT), *Thrips tabaci* (OT), and other thrips species encountered in various crops during a survey conducted across 8 large commercial floriculture greenhouses in Southern Ontario in summer 2016. The average proportion of each species across all sampling dates is presented, and the standard error (SE) was calculated based on the number of sampling events. “Other” thrips predominately include *Echinothrips americanus*, along with *Frankliniella fusca* and occasional specimens of *Anaphothrips obscurus* (less than 1% of all samples). Where OT represent more than 25% of the thrips species presented, data are bolded. Total species proportions and SE for the entire sampling period were calculated by averaging 12 crop/site averages.

Site	Crop	#of Dates	Total Thrips	WFT ± SE	OT ± SE	Other ± SE
1	Chrysanthemum (potted)	5	258	97.0% ± 1.7	3.0% ± 1.7	0.0% ± 0.0
2	Mandevilla	3	310	97.2% ± 1.4	1.4% ± 1.4	1.4% ± 1.4
	Gerbera (potted)	4	194	8.3% ± 2.5	**89.8% ± 4.4**	1.9% ± 1.9
3	Chrysanthemum (potted)	6	207	72.6% ± 13.5	**27.4% ± 13.5**	0.0% ± 0.0
	Hibiscus	7	299	99.4% ± 0.4	0.6% ± 0.4	0.0% ± 0.0
4	Gerbera (potted)	3	423	17.5% ± 8.0	**82.5% ± 8.0**	0.0% ± 0.0
5	Chrysanthemum (cut)	6	292	86.4% ± 5.7	12.8% ± 5.8	0.8% ± 0.6
6	Chrysanthemum (cut)	5	177	61.8% ± 18.3	5.2% ± 3.2	33.0% ± 20.1
	Gerbera (cut)	4	240	53.9% ± 14.3	**45.1% ± 14.2**	1.0% ± 0.6
7	Ivy Geranium	2	36	90.0% ± 10.0	10.0% ± 10.0	0.0% ± 0.0
	New Guinea Impatiens	4	165	83.4% ± 8.2	16.6% ± 8.2	0.0% ± 0.0
8	Chrysanthemum (potted)	2	62	1.0% ± 1.0	**99.0% ± 1.0**	0.0% ± 0.0
	**Total, all sites and crops**		**2663**	**59.1% ± 10.8**	**37.9% ± 3.2**	**3.2% ± 2.6**

**Table 3:** Thrips species causing outbreaks or unusual damage in greenhouse ornamentals since 2016, excluding western flower thrips. Thrips samples were collected by the authors or IPM consultants (indicated with asterisk) from greenhouses in Ontario. Species ratios are not reported, since only the thrips/crop of interest was the focus.

Species	Crop	Date
*Bagnalliella yuccae* (Hinds)	Yucca spp. *	December 2023
*Dichromothrips corbetti* (Priesner)	Phalaenopsis orchid	July 2023
*Echinothrips americanus* Morgan	Gerbera (cut)	November 2018
	Philodendron *	November 2022
*Frankliniella fusca* (Hinds)	Cyclamen	October 2018
	Cyclamen	October 2022
*Frankliniella schultzei* (Trybom)	Cactus *	November 2022
*Gynaikothrips uzeli* (Zimmermann)	Ficus benjamina *	November 2022
*Hercinothrips femoralis* (Reuter)	Unspecified ornamentals *	January 2017
	Gerbera (cut)	August 2018
	Spring bedding plants	February 2019
	Peperomia *	December 2022
*Scirtothrips dorsalis* Hood	Schefflera	December 2017
*Thrips nigropilosus* Uzel	Cyclamen	October 2018
	Chrysanthemum (potted)	August 2020
*Thrips parvispinus* (Karny)	Mandevilla/Dipladenia *	October 2021
	Mandevilla/Dipladenia *	February 2022
	Unspecified tropical ornamentals *	July 2022
*Thrips setosus* Moulton	Hydrangea *	April 2022
	Hydrangea *	October 2022
*Thrips tabaci* Lindeman	Osteospermum	May 2016
	Gerbera (potted)	July 2017
	Chrysanthemum (cut)	July 2021
	Cyclamen	October 2022
	Cyclamen	November 2022
	Anthurium *	November 2022
	Primrose	January 2023

## Data Availability

Data are available on request from the corresponding author (sarah.jandricic@ontario.ca).
